# Editorial: Present and future of EMDR in clinical psychology and psychotherapy, volume III

**DOI:** 10.3389/fpsyg.2025.1581456

**Published:** 2025-05-14

**Authors:** Antonio Onofri, Michael Hase

**Affiliations:** ^1^School of Specialization in Psychotherapy “Training School”, Rome, Italy; ^2^EMDR Center, Luneburg, Germany

**Keywords:** EMDR, psychotherapy, therapeutic process, PTSD, therapeutic relationship

In the evolving landscape of mental health treatment, Eye Movement Desensitization and Reprocessing (EMDR) therapy continues to stand out as one of the most transformative therapeutic approaches. Building on the foundations laid in the previous two volumes (Castelnuovo et al., 2019; Onofri, 2023), this third installment of *Present and Future of EMDR in Clinical Psychology and Psychotherapy* advances both theoretical understanding and clinical applications of EMDR therapy, expanding its scope beyond traditional trauma treatment.

Volume I, published in 2017, introduced a wide array of clinical evidence, showcasing EMDR therapy's effectiveness across conditions such as PTSD (Yurtsever et al., 2018; Wilson et al., 2018; Moreno-Alcázar et al., 2017), panic (Horst et al., 2017), depression (Hase et al., 2018; Ostacoli et al., 2018), somatic disorders (Szpringer et al., 2018) and other conditions (Carletto et al., 2018; Valiente-Gómez et al., 2017).

Volume II, released in 2023, highlighted EMDR therapy's adaptability also during the COVID-19 pandemic and its integration with other therapeutic modalities (Lazzaroni et al., 2021, 2022; Faretta et al., 2022; Yurtsever et al., 2022; Fernandez et al., 2022; Farrell et al., 2022). Together, these works have underscored the sustained growth and innovative evolution of EMDR.

This third volume reflects a further understanding of EMDR therapy's mechanisms, emphasizing its flexibility and broad applicability.

As we introduce the third volume of *Present and Future of EMDR in Clinical Psychology and Psychotherapy*, we continue to witness significant advancements in the application and understanding of Eye Movement Desensitization and Reprocessing (EMDR) therapy. In recent years, research has proliferated, demonstrating the efficacy of EMDR therapy in addressing trauma (Wright et al., 2024; Torres-Giménez et al., 2024; Matthijssen et al., 2024), panic (Inci Izmir et al., 2024), eating disorders (Rossi et al., 2024), mood disorders (Seok and Kim, 2024), psychosis (Varese et al., 2024; Marlow et al., 2024; Every-Palmer et al., 2024; Strelchuk et al., 2024), chronic pain (Vock et al.), and various other psychological conditions (Rodríguez-Garay and Mosquera, 2022; Martínez-Fernández et al., 2024; Schipper-Eindhoven et al., 2024; Zat Çiftçi et al., 2024; Stingl et al., 2024; Bal and Kiriş, 2024; Hafkemeijer et al., 2024). This volume reflects the ongoing commitment of the EMDR community to explore new clinical areas and expand our understanding of its therapeutic potential.

While previous volumes have made substantial contributions validating the effectiveness of EMDR therapy and uncovering its underlying mechanisms (Landin-Romero et al., 2018; Pagani et al., 2017; Matthijssen et al., 2017; Hase et al., 2017), it is becoming increasingly important that future research also turns its focus toward the therapeutic process itself (Ramallo-Machin et al.). Understanding how EMDR unfolds in real-time within the therapeutic relationship (Hase and Brisch, 2022), and what factors contribute to its success beyond mechanistic or outcome-driven research, could illuminate pathways to more individualized and effective interventions. In addition this volume reflects the development of the EMDR technique to the comprehensive psychotherapy which EMDR therapy is.

The articles included in this volume mark a decisive step forward in this direction.

More than one contribution, for example, emphasize the importance of therapist-patient attunement, the nuances of countertransference, and the role of emotional resonance during sessions (Ramallo-Machin et al.; Hase et al.). These elements remind us that EMDR is not merely a technique applied in isolation but part of a dynamic interaction where the relational and emotional context is critical, as it is EMDR therapy.

Looking ahead, we must encourage future research to delve deeper into these relational processes. By studying not only *what* makes EMDR therapy effective but *how* it works within the therapeutic alliance, we will be better positioned to refine our approaches and enhance the quality of care. A broader inquiry into the therapist's emotional regulation, sensitivity to patient cues, and the co-creation of a safe therapeutic space could offer invaluable insights.

Additionally, while the drive to reveal the neurobiological mechanisms of EMDR therapy has so far provided essential data, the complexity of the therapeutic process cannot be fully captured by brain scans or quantitative measures alone. We must balance the quest for scientific validation with a richer understanding of the lived experiences of both patients and therapists within the EMDR therapy framework. Future studies should aim to include qualitative methods that explore the subjective, emotional, and interpersonal dimensions of EMDR therapy, thus complementing the current focus on empirical outcomes.

This volume surely underscores the continuing evolution of EMDR therapy as a clinical tool, and it is an honor to present the contributions of distinguished researchers and practitioners. As we look toward the future, let us prioritize not only the technical refinement of EMDR therapy but also a deeper exploration of the relational and emotional processes that make it such a profoundly transformative therapy.

## Acknowledging EMDR Europe's role in advancing research

Before delving into the key contributions of this volume, we wish to recognize the critical role that EMDR Europe has played in fostering research and clinical excellence. Their continuous commitment to advancing empirical research has driven significant progress in the development of EMDR therapy treatment plans, often referred to as protocols. Many recent research owe much to their support, which has fortified the scientific underpinnings of EMDR therapy, securing its position as an evidence-based approach to trauma and beyond.

## Rapid growth of EMDR research

The body of research surrounding EMDR therapy is growing rapidly, with a notable shift in geographical focus. While the United States has historically been the center of EMDR therapy research in the beginning, Europe has emerged as a significant contributor. Countries such as the Netherlands, Spain, the UK, Italy, and Germany are leading the way, advancing the field through robust research initiatives. Moreover, emerging states are beginning to explore and implement EMDR therapy, signaling a broader international interest and EMDR therapy's expanding reach.

## Key contributions of volume III

This volume features a range of contributions that deepen our understanding of EMDR therapy's applications. The articles exemplify the versatility and impact of EMDR therapy, from its integration with attachment theory to novel applications in pediatric oncology. A brief overview of the four main articles in this volume follows:

1. ***The Therapeutic Relationship in EMDR Therapy - A survey*** (Hase et al. 2024): This study revisits the foundations of EMDR therapy's development, particularly the evolution of the Adaptive Information Processing (AIP) model, and explores the role of the therapeutic relationship within an attachment framework. Based on a modified Delphi method, the study gathered insights from EMDR therapists to assess the attachment-based concept of the therapeutic relationship in EMDR therapy. Results support this perspective, highlighting the importance of the therapeutic bond, and suggesting implications for training and clinical application.
2. ***Apples and Oranges: Comparing PTSD Patients and Healthy Individuals in Bilateral Stimulation Responses*** (Pape et al. 2024): This study investigates physiological and subjective responses to bilateral stimulation (BLS) among PTSD patients compared to healthy individuals. Findings indicate distinct differences: while healthy individuals displayed reduced distress and enhanced attention under BLS, PTSD patients showed subjective relief without corresponding physiological changes. These results caution against assuming that BLS responses in healthy populations are directly transferable to clinical PTSD contexts.
3. ***Factors Influencing Quality of Processing in EMDR Therapy*** (Ramallo Machin et al. 2024): This article introduces the Processing Difficulties Scale (PDS), developed to identify and address in-session processing challenges specific to EMDR therapy. The study highlights the impact of complex trauma, dissociative symptoms, and emotional dysregulation on processing effectiveness, particularly in clients with early traumatization. This new scale offers a promising tool for optimizing therapeutic outcomes by tailoring interventions to address processing styles and challenges effectively.
4. ***EMDR in Pediatric Psychology: A Case Report of an Adolescent with Cancer*** (Zucchetti et al. 2024): A groundbreaking case study explores the application of EMDR therapy in treating PTSD symptoms in a 17-year-old cancer patient, a novel approach in pediatric oncology. The case demonstrates the EMDR therapy protocol's effectiveness in reducing emotional distress related to a leukemia diagnosis, supporting its potential as an early intervention in pediatric oncology settings.

## Looking toward the future: EMDR therapy's expanding frontiers

As this third volume reveals, the future of EMDR therapy continues to unfold in promising directions. Innovations such as the Processing Difficulties Scale offer new insights into optimizing EMDR therapy sessions, particularly for complex trauma cases. With growing interest in integrating EMDR therapy with cutting-edge technologies, there is immense potential to create more immersive therapeutic experiences. Additionally, emerging research suggests EMDR therapy's applicability for somatic conditions like chronic pain, representing a new frontier in mind-body approaches.

## Conclusion

The research compiled in *Present and Future of EMDR in Clinical Psychology and Psychotherapy, Volume III* reflects the ongoing growth and innovation within EMDR therapy. Building on the solid foundations of Volumes I and II, this volume brings fresh perspectives and expands the evidence base for EMDR therapy's use in diverse settings and populations. Thanks to EMDR Europe's steadfast support in the field of research, the field is moving toward higher scientific rigor and broader accessibility, ensuring that EMDR therapy will continue to play a vital role in trauma-informed care and in psychotherapy in general worldwide for years to come.
